# Direct-writing of PbS nanoparticles inside transparent porous silica monoliths using pulsed femtosecond laser irradiation

**DOI:** 10.1186/1556-276X-6-542

**Published:** 2011-10-04

**Authors:** Abdallah Chahadih, Hicham El Hamzaoui, Rémy Bernard, Luc Boussekey, Laurence Bois, Odile Cristini, Marc Le Parquier, Bruno Capoen, Mohamed Bouazaoui

**Affiliations:** 1 Laboratoire de Physique des Lasers, Atomes et Molécules (CNRS, UMR 8523), IRCICA (USR CNRS 3380), CERLA (FR CNRS 2416), Bâtiment P5, Université Lille 1-Sciences et Technologies, F-59655 Villeneuve d'Ascq Cedex, France; 2 Laboratoire de Spectrochimie Infrarouge et Raman CNRS UMR 8516 USTL Bât C5, Université Lille 1-Sciences et technologies 59655 Villeneuve d'Ascq Cedex, France; 3 Laboratoire des Multimatériaux et Interfaces (CNRS, UMR 5615), Bâtiment Berthollet, 22 Avenue Gaston Berger, Université de Lyon 1, 69622 Villeurbanne Cedex, France

## Abstract

Pulsed femtosecond laser irradiation at low repetition rate, without any annealing, has been used to localize the growth of PbS nanoparticles, for the first time, inside a transparent porous silica matrix prepared by a sol-gel route. Before the irradiation, the porous silica host has been soaked within a solution containing PbS precursors. The effect of the incident laser power on the particle size was studied. X-ray diffraction was used to identify the PbS crystallites inside the irradiated areas and to estimate the average particle size. The localized laser irradiation led to PbS crystallite size ranging between 4 and 8 nm, depending on the incident femtosecond laser power. The optical properties of the obtained PbS-silica nanocomposites have been investigated using absorption and photoluminescence spectroscopies. Finally, the stability of PbS nanoparticles embedded inside the host matrices has been followed as a function of time, and it has been shown that this stability depends on the nanoparticle mean size.

## Introduction

New directions of modern research have emerged during the last decade, which has broadly been defined as nanoscale science and technology [1,2]. These new trends involve the ability to fabricate, characterize, and manipulate artificial structures, the features of which are controlled at the nanometer scale. The semiconductor properties of lead sulfide PbS have widely been used in elements such as detectors operating in the infra-red spectral region (from 850 to 3100 nm) [3], solar batteries [4], and advanced optoelectronic devices [5]. In most of recent applications, useful properties of the PbS nanoparticles arise from the strong quantum confinement effect of the charge carriers (Bohr radius of 18 nm) and the associated optical nonlinear property [6].

Semiconductors may be included in a variety of media including polymers [7-9], micelles [10,11], glassy particles [12], and zeolites [13]. The synthesis of semiconducting nanoparticles in glass matrices has become very important. A number of methods to synthesize semiconductor nano-crystals have been employed and these include hydrothermal synthesis [14], chemical bath [15], spray pyrolysis [16], laser-heated evaporation [17], combustion synthesis [18], and the sol-gel technique [19]. The sol-gel technique has become very popular because of the high chemical homogeneity of the products, also to the low processing temperatures, the possibility of controlling the particles size and morphology. The sol-gel-derived materials provide excellent matrices for a variety of organic and inorganic compounds. Silica porous matrices can be obtained using the sol-gel process and depending on how the wet gel is dried, the porosity of the materials can be tailored [20]. Such a controlled porosity can be used to dope it with active elements by impregnation with appropriate solutions.

A few articles have been reported in the literature on the crystallization of PbS nanoparticles by heat-treatment inside a silica matrix prepared by sol-gel method [21-23]. In this case, however, the precipitation of semiconductor nanoparticles has been realized without any space-localization. To reach this aim, the use of a laser irradiation is a promising method. Recently, local precipitation of PbS nanoparticles in melting glass matrix has been reported [24,25]. Chao et al. [24] used pulsed femtosecond laser irradiation (800 nm, 1 kHz, and 184 fs) followed by a heat treatment at a temperature of 450°C to precipitate PbS nanoparticles inside irradiated areas of a melted glass. Takeshima et al. [25] reported the local precipitation of PbS inside a melted glass without annealing using a pulsed femtosecond laser irradiation at high repetition rate (250 kHz, 200 fs, 750 mW). Recently, we have reported the space-selective growth of CdS nanoparticles, at ambient conditions, inside silica xerogels by laser irradiation [26].

Herein, we report on our investigations, based on a pulsed femtosecond laser irradiation performed with a low repetition rate in ambient conditions, for the localized growth of PbS nanoparticles inside a deep volume of porous silica monoliths. Moreover, we provide a study on the stability of PbS nanoparticles as a function of time.

## Experimental

Porous silica xerogels with a thickness of 2 mm has been prepared by a sol-gel route using tetramethylorthosilicate [20]. The obtained silica monolith exhibited interconnected pores of mean diameter 5.4 nm, a specific surface area of 360 m^2 ^g^-1 ^and a pore volume of 0.49 cm^3 ^g^-1^. All these parameters were determined by isothermal nitrogen sorption measurements, as previously described [20]. The obtained porous silica samples were impregnated for 4 h into an aqueous solution (S) composed of the lead acetate as a lead precursor and of thiourea as a sulfur precursor. The concentration of each precursor in S was 0.37 mol L^-1 ^and the molar ratio between Pb and S sources was kept to 1.

**Figure 1:**
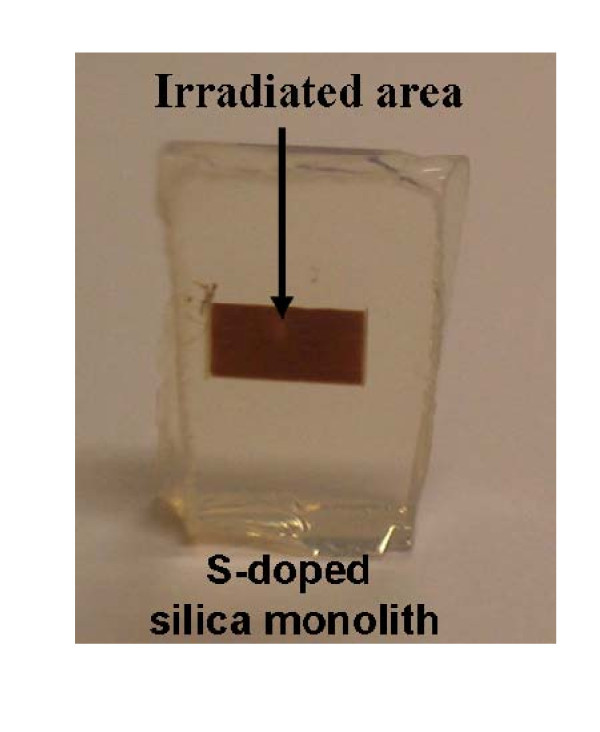
**The irradiated area (10 mW) inside an S-doped silica monolith**.

The irradiation experiments were performed using a Ti:sapphire oscillator, followed by a regenerative amplifier producing 120 fs pulses at 800 nm with a 1 kHz repetition rate. The laser beam, with an average power ranged between 10 and 40 mW, was focused through a 10× microscope objective with a numerical aperture of 0.30. The obtained spot, of a diameter estimated to 2 μm, was located inside the volume of the silica matrix and scanned laterally at a rate of 1 mm s^-1^. A tight network of irradiated lines with a step size of 20 μm has been performed to cover a wide zone. The colored area was then large enough to be observed with the naked eye and to be optically characterized. Figure 1 of the S-loaded silica monolith shows the irradiated area inside the deep volume of the xerogel for a laser power of 10 mW, corresponding to a crest irradiance of about 2.5 × 10^15 ^W cm^-2^.

Absorption spectra were recorded at room temperature in the irradiated zone using a Perkin-Elmer lambda 19 UV-Vis-IR double beam spectrometer.

Micro-photoluminescence measurements have been performed using a HORIBA Jobin Yvon IHR320 spectrometer coupled with a microscope at room temperature. Both 351.1 and 514.5 nm laser lines of an argon laser have been used to excite the PbS-doped silica matrices in the fs-irradiated zone. The incident laser power was kept low enough to avoid any further precipitation of PbS nanoparticles.

XRD patterns are recorded on a Philips X'Pert diffractometer equipped with a monochromator, using Cu Kα radiation.

TEM characterization was performed on a microscope FEI Tecnai G2-20 twin with a 200 kV acceleration voltage. For TEM imaging, the precipitated zone of a doped sample was grinded into a powder and then deposited onto a copper grid previously coated with a thin carbon membrane. The powder was then metalized with a vaporized carbon layer.

## Result and discussion

The irradiation wavelength 800 nm corresponds to energy much smaller than the band gap of silica. Hence, no linear absorption can occur when the matrix is irradiated, thus allowing the laser beam to penetrate deeply inside the material. Hence, it is possible to embed a PbS layer of nanoparticles anywhere in the monolith. However, even at the lowest average power corresponding to tremendous laser peak power, a multi-photon absorption exists. As a result, the impact area is highly localized, confined within the focal volume of the focused beam. The irradiated zone showed brown or black color, depending on the average power of the laser beam. This color is easily visible to the naked eye, as shown in Figure 1.

The formation of PbS nanocrystals inside the porous silica glass, after laser irradiation, was confirmed by different analysis techniques. According to the different analysis techniques, we have irradiated at different depths: just under the surface for XRD measurements and for the other characterisations, the focused beam was in the middle of the monolith.

**Figure 2:**
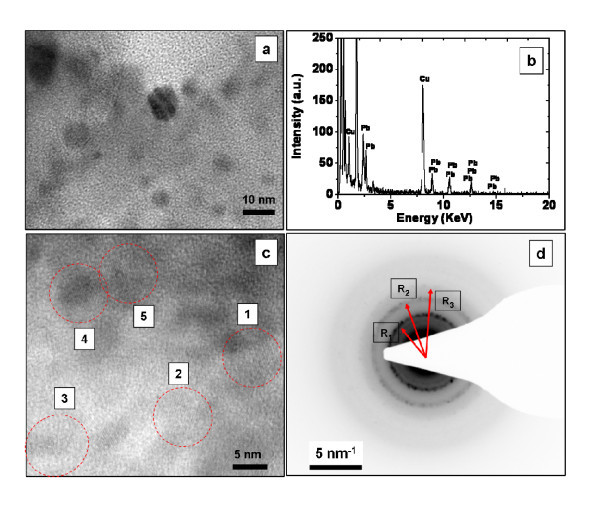
**Results of TEM measurements in the irradiated area with 40 mW**: **(a) **TEM image, **(b) **EDX spectrum obtained from an ensemble of PbS nanocrystals, **(c) **HR-TEM image of several PbS nanoparticles, **(d) **electron diffraction image taken from a zone filled with nanoparticles.

TEM analysis has been performed to evidence the formation of PbS nanoparticles. Figure 2a shows a TEM image of the irradiated area at 40 mW. Quasi-spherical nanoparticles, with a size distribution ranging between 5 and 12 nm, have been observed. Figure 2b shows the EDX analysis recorded on the same area as in Figure 2a, confirming that the obtained nanoparticles contained Pb atoms. The Cu peaks at approximately 1 and 8 keV came from the TEM grid. Moreover, Figure 2c presents the HR-TEM of different nanoparticles. The calculation of the atomic interplanar distances has been performed on five zones labeled by circles. The zones 1, 2, and 3 exhibit fringe distances around 0.34 nm, which are attributed to the (111) lattice planes of cubic PbS (*d*_111 _= 0.343 nm, JCPDS card, reference code 03-065-0692). While the fringes spacing for the zones 4 and 5 were of about 0.3 nm, which corresponds to the (200) lattice planes of PbS (*d*_200 _= 0.297 nm, JCPDS card, reference code 03-065-0692). In addition, Figure 2d presents an electron diffraction pattern taken in a zone filled with nanoparticles. The estimated diffraction radii R1, R2, and R3 correspond well to the PbS lattice planes (200), (220), and (222), respectively.

**Figure 3:**
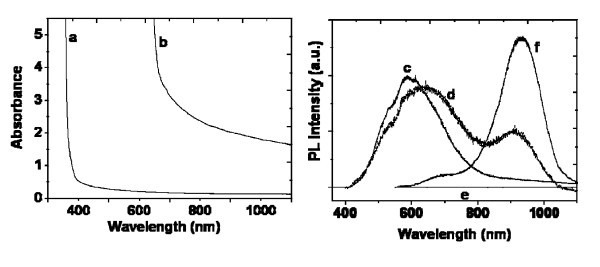
**Left: Optical absorbance spectra of S-doped silica matrix**: **(a) **non-irradiated area, **(b) **femtosecond laser irradiated area with 40 mW. Right: **(c) **PL spectrum of a non-irradiated area under excitation at 351 nm, **(d) **PL spectrum of the irradiated area under excitation at 351 nm, **(e) **PL spectrum of a non-irradiated area under excitation at 514.5 nm and **(f) **PL spectrum of the irradiated area under excitation at 514.5 nm.

Optical absorption spectra have been recorded in the irradiated and in the non-irradiated areas, as shown in Figure 3(left). The absorption spectrum of the zone irradiated with an average power of 40 mW is red-shifted as compared to the absorption spectra of the non-irradiated zone. Moreover, one can note that the absorbance baseline in the irradiated area is much higher than those of the non-irradiated zone. Such a strong absorption level has been attributed to the formation of PbS nanoparticles in high concentration. The absence of any excitonic absorption structure in the absorption spectra may have two main reasons: first, the weak exciton binding energy because of the strong Coulomb screening in narrow gap semiconductors and second, the existing size distribution of the nanocrystallites.

Photoluminescence spectra of the area irradiated with a power of 40 mW and in the unirradiated area are shown in Figure 3(right). Under excitation with the 351 nm laser line, the non-irradiated area emits light in a large band centered on 600 nm, while the excitation of the irradiated area at the same wavelength results in two emission bands: the first one being centered on 650 nm and the second one around 910 nm. Consequently, the bands peaking at 600 and 650 nm could be attributed to the luminescence of PbS precursors and SiO_2 _host matrix, since the S-doped silica absorption at 350 nm is still strong. On the other hand, the PL band centered at 910 nm is due to the PbS nanoparticles. To confirm our assumption, the same PL spectra were recorded under excitation at 514.5 nm. At this wavelength, the S-loaded matrix absorbs less and as a result, no emission could be observed in the non-irradiated area. However, in the case of the irradiated area, both emission bands are still present, although red-shifted by 20 nm. As far as the PbS nanoparticles are concerned, the red-shift of the 650 nm emission band can be interpreted through a resonant size-selection by the excitation wavelength, as already observed with CdS particles [27]. The same kind of selection process in the electronic level population may be assumed for the "matrix band" at 650 nm. Meanwhile, the intensity ratio between these two emission bands is inverted when switching from UV to visible excitation. This can easily be understood as the result of a drastic decreasing number of absorbing entities in the non-irradiated zone between 351 and 514 nm wavelengths. The maximum of the emission band located around 930 nm is because of the presence of dominant small PbS nanoparticles (3-5 nm) [28]. This maximum emission is shifted toward lower wavelengths comparing to the one reported by Hines and Scholes [29] for a bigger colloidal PbS with a size of 6.5 nm.

In the literature, the mechanism of PbS nanoparticles formation inside a porous silica matrix has been explained by pyrolysis or decomposition of the PbS precursor under heat treatment [21,22]. Moreover, Chao et al. [24] have reported on the mechanism of PbS nanoparticles precipitation inside melting glasses induced by a femtosecond laser: when glasses containing lead and sulfur are submitted to a femtosecond laser irradiation, it can probably induce ion-exchange effects and a redistribution of network modifiers, resulting in the enrichment of the laser-irradiated zone in lead and sulfur atoms. This enrichment makes it possible to decrease the thermal treatment temperature. Hence, the formation of bigger and denser quantum dots in the irradiated area is accelerated after a subsequent and necessary heat treatment. In our case, the nano-crystallization of PbS inside the porous silica occurred in the region of the laser irradiation without any further annealing because of two main processes. First, the multi-photon absorption of the porous silica matrix and of the PbS precursors at 800 nm [30] leads to the decomposition of these precursors, thus enriching the irradiated zone with lead and sulfur ions. In the same time, the temperature elevation during the laser pulse could be sufficient to aggregate the molecular PbS into nanoparticles [26,31].

**Figure 4:**
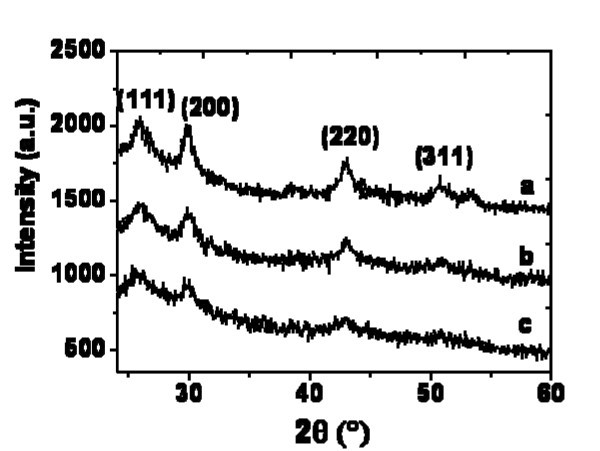
**XRD patterns of S-doped silica matrices recorded in the irradiated area with different laser powers**: **(a) **40 mW, **(b) **25 mW and **(c) **10 mW.

The effect of the incident average laser power on the size of the PbS nanocrystallites has been studied. Three S-doped samples were irradiated by the pulsed femtosecond laser at three different incident powers: 10, 25, and 40 mW. XRD measurements were performed on the irradiated samples, as shown in Figure 4. For each power, the XRD pattern presents reflections for 2θ equal to 26.1°, 29.9°, 42.9°, and 50.8°. These reflexes can be attributed to (111), (200), (220), and (311) reticular planes of the PbS cubic phase, respectively [21,22]. One can note an increase in intensity and a decrease in width of the reflexes with increasing the irradiation power. The mean nanocrystal diameter, *d*, can be determined from the line width of the XRD pattern by the Scherrer formula [32].

(1) $$d=\left(0.94\lambda \right)∕\left(B\cos\theta_{B}\right)$$

where λ is the X-ray wavelength, *B *is the full width at half maximum of the diffraction reflex (in radian), and *θ_B _*is the half angle position of the diffraction reflex on the 2*θ *scale. The diameter of the PbS nanoparticles, calculated from the Scherrer formula (Equation 1), is summarized in Table 1.

**Table 1:** Calculated sizes of PbS nanoparticles from XRD measurements for different laser average powers

Power (mW)	Sizes calculated from XRD (nm)
10	4
25	7
40	8

The most important drawback that limits the use of PbS nanoparticles in many applications is their low chemical stability. This is due to their high ability to oxidation, which limits their use under ambient conditions.

**Figure 5:**
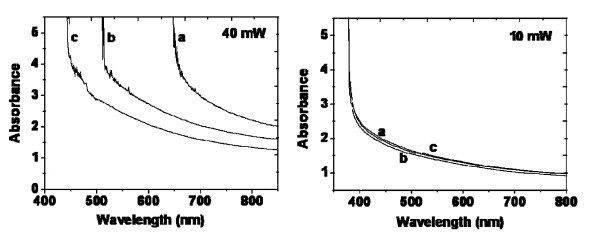
**Optical absorption of PbS nanoparticles created by femtosecond laser at different times**. Left: sample irradiated with 40 mW, Right: sample irradiated with 10 mW. **(a) **100 days after the initial irradiation, **(b) **50 days after the initial irradiation and **(c) **just after the initial irradiation.

To indirectly investigate the stability of PbS nanoparticles precipitated inside the silica host matrix by pulsed laser irradiation, irradiated samples have been left in ambient air a for long period and optical absorption spectrum has been monitored upon time. Figure 5 shows the absorption spectra of two samples irradiated with 10 and 40 mW at three different times after the irradiation. One can note that the absorption edge of the sample irradiated with 40 mW was blue-shifted after 100 days. This blue-shift is attributed to the oxidation of PbS nanoparticles created inside the silica matrix. On the other hand, the absorption edge of the sample irradiated with 10 mW presents no shift after 100 days in comparison with the absorption taken just after preparation.

These preliminary results demonstrate that the stability of PbS nanoparticles, inside the porous host matrix, depends on their size. It should be recalled that the PbS particle mean size, as deduced from the XRD measurements around time t_0_, has been evaluated to 4 and 8 nm after irradiations with 10 and 40 mW, respectively (see Table 1). In the first case, the crystallite size is thus lower than the average matrix pore size of 5.4 nm, as opposed to the case of 40 mW irradiation. Since the diameter size of PbS nanoparticles created with 40 mW is greater than the pore diameter, it implies that, under the extreme local conditions of the femtosecond laser-induced plasma, the created nanoparticles are capable of breaking the walls of the interconnected pores during their formation. An important amount of oxygen is then allowed to diffuse inside the matrix and to react with PbS nanoparticles, leading to their oxidation in several days. It has been noticed that the oxidation rate seems to decrease versus time. This shift in the absorption band edge could be explained by the formation of core-shell structure with unknown shell phase. Indeed several oxide phases can be observed when PbS is oxidized such as PbO. PbSO_4 _[33]. We have already detected this phase when we irradiate the doped monoliths by 514.5 nm continuous laser. On the contrary, no oxidation was observed with a lower laser power of 10 mW, but most of the nanoparticles have a size (4 nm) suited to the pores. In this case, it is likely that the nanoparticles have grown without any modification of the silica matrix and these particles remain protected from oxidation by the silica walls. This effect could explain the stability of PbS nanoparticles, even after 100 days. Hence, both the pore diameter of the host silica matrix and the power of pulsed femtosecond laser irradiation play an important role in the stability of the obtained PbS nanoparticles.

## Conclusion

The formation of PbS nanoparticles localized inside porous silica xerogels has been performed using the pulsed femtosecond laser irradiation without any annealing. Characterization of the obtained nanocrystals was allowed by writing a tight network of PbS lines in a centimeter-sized zone near the surface or in the depth of the bulk xerogel. Most of the crystallites have been identified as cubic phase of PbS by XRD and TEM. We have shown that the power laser can be used to control the mean particle size between 4 and 8 nm. The micro-PL data made it possible to identify an emission band coming from the matrix and PbS precursors, and another band assigned to PbS nanoparticles, which are resonantly wavelength-selected in size. Finally, the stability of PbS nanoparticles under ambient conditions depends on the particle size, monitored by the laser power, and on the porosity of the host matrix.

## Abbreviations

PbS: lead sulfide; PL: photoluminescence; XRD: X-ray diffraction.

## Competing interests

The authors declare that they have no competing interests.

## Authors' contributions

AC and HE carried out the preparation of the samples as well as the writing of the manuscript. RB and LB have preformed the XRD analysis and the calculation of sizes. LB carried out the PL analysis. MLP is responsible for the femtosecond laser operations. AC, HE, OC, BC, and MB have interpreted the results, as well as drafted the manuscript. All authors read and approved the final manuscript.
